# Human Adipose-Derived Mesenchymal Stromal Cells Exhibit High HLA-DR Levels and Altered Cellular Characteristics under a Xeno-free and Serum-free Condition

**DOI:** 10.1007/s12015-021-10242-7

**Published:** 2021-09-11

**Authors:** Phuong T. M. Dam, Van T. Hoang, Hue Thi Hong Bui, Le Minh Hang, Duc M. Hoang, Hoang Phuong Nguyen, Ha Thi Lien, Huong Thi Thanh Tran, Xuan-Hung Nguyen, Liem Nguyen Thanh

**Affiliations:** 1 Vinmec Institute of Applied Science and Regenerative Medicine, Vinmec Health Care System, Hanoi, Vietnam; 2 Vinmec Research Institute of Stem Cell and Gene Technology (VRISG), Vinmec Health Care System, Hanoi, Vietnam

**Keywords:** Adipose-derived stem cells, Adipose-derived mesenchymal stem cells, Adipose-derived mesenchymal stromal cells, HLA-DR, Karyotype, Senescence

## Abstract

**Background:**

We have observed an increased expression of negative markers in some clinical-grade, xeno- and serum-free cultured adipose-derived mesenchymal stem/stromal cell (ADMSC) samples. It gave rise to concern that xeno- and serum-free conditions might have unexpected effects on human ADMSCs. This study aims to test this hypothesis for two xeno- and serum-free media, PowerStem MSC1 media (PS) and StemMACS MSC Expansion Media (SM), that support the in vitro expansion of ADMSCs.

**Methods:**

We investigated the expression of negative markers in 42 clinical-grade ADMSC samples expanded in PS. Next, we cultured ADMSCs from seven donors in PS and SM and examined their growth and colony-forming ability, surface marker expression, differentiation, cell cycle and senescence, as well as genetic stability of two passages representing an early and late passage for therapeutic MSCs.

**Results:**

15 of 42 clinical-grade PS-expanded ADMSC samples showed an increased expression of negative markers ranging from 2.73% to 34.24%, which positively correlated with the age of donors. This rise of negative markers was related to an upregulation of Human Leukocyte Antigen – DR (HLA-DR). In addition, the PS-cultured cells presented decreased growth ability, lower frequencies of cells in S/G2/M phases, and increased ß-galactosidase activity in passage 7 suggesting their senescent feature compared to those grown in SM. Although MSCs of both PS and SM cultures were capable of multilineage differentiation, the PS-cultured cells demonstrated chromosomal abnormalities in passage 7 compared to the normal karyotype of their SM counterparts.

**Conclusions:**

These findings suggest that the SM media is more suitable for the expansion of therapeutic ADMSCs than PS. The study also hints a change of ADMSC features at more advanced passages and with increased donor’s age. Thus, it emphasizes the necessity to cover these aspects in the quality control of therapeutic MSC products.

**Graphical abstract:**

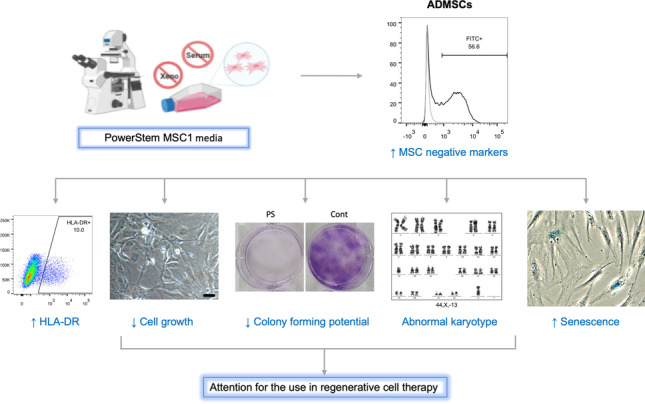

**Supplementary Information:**

The online version contains supplementary material available at 10.1007/s12015-021-10242-7.

## Introduction

Adipose-derived mesenchymal stem cells/stromal cells (ADMSCs) have great potential for cell-based therapy and regenerative medicine due to their capability of immunomodulation and in vitro differentiation into various cell lines such as adipocytes, chondrocytes, osteoblasts [1], neuronal cells [2, 3] and hepatocytes [4]. Additionally, growth factors, cytokines, exosomes and microvesicles secreted by MSCs also contribute to their regenerative activity in vivo [5–7]. Adipose tissue is an abundant source for MSCs in many clinical trials [8]. Compared to bone marrow-derived MSCs (BM-MSCs), the harvesting procedure of ADMSCs is less invasive and can be performed through multiple sites resulting in high stem cell yield [9]. ADMSCs have been applied to treat Crohn’s fistula in a phase III trial [10], osteoarthritis and degenerative eye diseases in phase II studies [11–13]. Of note, all these trials required an in vitro expansion of isolated ADMSCs to obtain sufficient cell doses.

Although there are several commercially serum-free and xeno-free media, the expansion of ADMSCs and their biological properties in these media remain elusive. MSCs might lose their growth and multipotency upon the in vitro culture [14, 15]. Indeed, ADMSC proliferation declined after five passages in a commercial xeno-free and serum-free media called StemPro (Invitrogen, Carlsbad, CA, USA) [16]. Culture of dental pulp stem cells and alveolar bone marrow MSCs in StemPro and StemMacs_MSC/XF (Miltenyi Biotec GmBH, Germany) resulted in reduced chondrogenic and adipogenic differentiation potential [17]. Furthermore, cardioprotective effects of MSCs diminished in a rat model for ischemia–reperfusion injury when cells of advanced passages (passage 5 to passage 10) were administrated [18]. Another challenge is that genetic changes such as tetrasomy and other chromosomal abnormalities might occur in MSC cultures [19, 20]. These can be related to spontaneous tumorigenic transformation and become a potential risk for MSC-based therapy [21]. In addition to in vitro related dysfunctions of MSCs, donor features also play an essential role in the biological activities of these cells. MSCs obtained from aged donors were associated with lower growth kinetics, increased senescence, and altered differentiation potential [22–24]. Metabolic diseases such as type 2 diabetes may restrict MSC functions [25, 26]. Therefore, the influence of culture conditions and donor characteristics on MSC characteristics should be addressed.

According to the guidance of the International Society for Cellular Therapy (ISCT), one of the criteria to define MSCs are the surface expression of CD73, CD90, CD105, CD44 and the absence of CD45, CD34, CD14/CD11b, CD19/CD79α, CD31, as well as the human leukocyte antigen HLA-DR [27, 28]. The last marker, HLA-DR, belongs to the major histocompatibility complex class II (MHC class II) and plays a crucial role in host immune responses. Allogenic MSCs were rejected in MHC class I- and MHC class II-mismatch recipient mice [29] and induce an immune response in pigs and horses [30–32]. Moreover, HLAs were demonstrated to induce the immunogenicity of allogenic ADMSCs [33]. This suggested that HLA-DR expression might induce an immune response and increase the risk of rejection in an allogeneic environment. For this reason, HLA-DR expression in cultured ADMSCs requires a special attention.

This study analyzed the immunophenotype of clinical-grade ADMSCs in a histological cohort, which were expanded under a serum-free and xeno-free condition using PowerStem MSC1 media (PS). Next, we compared the cellular properties of ADMSCs cultured in PS with their counterparts grown in our new candidate for serum-free and xeno-free medium called StemMACS MSC Expansion Media (SM).

## Materials and Methods

### Patient Samples

The adipose tissues were obtained at Vinmec International Hospital between 2018 and 2019 and cryopreserved in the biobank after the donors signed the informed consent form. The expression of surface markers was retrospectively analyzed on 42 ADMSC samples isolated in PS (Pan-Biotech, Germany), of which 31 patients participated in a previous study [34]. This study enrolled patients with sexual functional deficiency. The other 11 samples were obtained from healthy donors. We performed a prospective study on seven samples, of which donors agreed to donate their biological materials for the current research, to investigate the cellular properties of ADMSCs cultured in different xeno-free and serum-free MSC expansion media (Suppl. Figure 1). Sample collection and data analysis were approved by the Ethics Committee of Vinmec Healthcare System and carried out in accordance with the Declaration of Helsinki.

### Isolation, Culture, and Cryopreservation of ADMSCs

ADMSCs were isolated as previously described [35]. Briefly, adipose tissues were harvested from the donor’s lower abdomen under general anesthesia. Then adipose tissue was minced into small pieces and digested using 200 unit/ml Collagenase type I solution (Gibco, Denmark) supplemented with 200 g/l Human Albumin (Baxter, USA) at 37 °C with agitation 300 rpm/min for 60 min. The mixture was centrifuged at 1500 rpm/min for 10 min to collect the stromal vascular fraction in the pellet. Finally, the cells were resuspended in PS (Pan-Biotech, Germany) supplemented with 1% Penicillin/Streptomycin (Gibco, USA). Cells were incubated at 37 °C and 5% CO_2_ in a humidified atmosphere with medium exchange every three days. When reached 80–90% confluence, the cells were harvested using TrypLE™ Select Enzyme (Gibco, USA) and counted after staining with trypan blue (Life Technologies, USA).

Cells at passage 0 were cryopreserved in the Cryostor solution (Stemcell Technologies, Canada). All the cryovials were kept at -80 °C for no more than seven days and transferred to BioStore™ III Cryo (Brooks Life Sciences, USA) for long-term storage. After four months of cryopreservation, the cells were thawed rapidly at 37 °C and maintained in PS or SM (Miltenyi Biotec, Germany) to evaluate cell growth, phenotype, and functional properties.

### Growth Curve

To determine the proliferative capability of ADMSCs, cells were seeded with the density of 5,000 cells/cm^2^ in T25 flasks (Nunc) and cultured up to passage 7 as previously described [35]. Experiments were performed in triplicate for each donor. Population doubling (PD) was calculated using the formula: PD = 3.32 × log (a/b), in which a is the number of harvested cells and b is the seeding density. Finally, the population doubling time (PDT) was calculated as t/n, where t is the days (or hours) of culture, n is the number of PD reached during a passage. The cell morphology was visualized under an invert phase-contrast microscope (Eclipse Ti-S/DS-Fi2-L3, Japan).

### Colony-Forming Unit (CFU) Assay

Cells were seeded in triplicate in a six-well dish at the concentration of 20 cells/cm^2^ and cultured in the respective media (PS or SM) for 10 to 14 days as previously described [35]. Cells were fixed in Methanol (Merck, Germany) and stained with Giemsa solution (Merck, Germany), both for 5 min at room temperature. Colonies formed on each dish were counted.

### Flow Cytometry Analysis

ADMSCs harvested from passage 3 and 7 were assessed for surface marker expression by flow cytometry. Cells were resuspended in PBS (Gibco, USA) supplemented with 1% human serum (Pan-Biotech, Germany). The cells were incubated using the Human MSC Analysis kit (Becton Dickinson, USA) according to the manufacturer’s instruction. To examine the expression of negative surface markers individually, cells were stained with the following antibodies: CD34 Monoclonal Antibody (581) FITC (Invitrogen, USA), CD11b Monoclonal Antibody (ICRF44) PE (Invitrogen, USA), CD19 Monoclonal Antibody (HIB19) PerCP-Cyanine 5.5 (Invitrogen, USA), CD45 Monoclonal Antibody (HI30) APC (Invitrogen, USA), Anti HLA-DR-FITC (Beckman Coulter, France). The samples were analyzed using BD Canton II (Becton Dickinson, USA). At least 20,000 cells per run were acquired. The data analysis was performed using the FlowJo software (Becton Dickinson, USA).

To check viability, cells were suspended in PBS (Gibco, USA) supplemented with 1% human serum (Pan-Biotech, Germany) and stained with 7-AAD Staining Solution (Miltenyi, Germany) following the manufacturer’s instruction. For the cell cycle analysis, cells were fixed with eBioscience™ Intracellular Fixation & Permeabilization Buffer Set (Invitrogen/Thermo Fisher Scientific, USA) and stained with 7-AAD Staining Solution (Miltenyi, Germany) as previously described [36]. At least 20,000 cells were analyzed per run using BD Canton II (Becton Dickinson, USA) and the FlowJo software (Becton Dickinson, USA).

### MSCs Differentiation Assays

MSCs were differentiated into adipogenic, chondrogenic and osteogenic lineages using the StemPro® Adipogenesis Differentiation Kit, StemPro™ Chondrogenesis Differentiation Kit, StemPro™ Osteogenesis Differentiation Kit (Gibco, USA) in duplicates as previously described [35]. For this purpose, cells were cultured in either differentiation media or MSC culture media as the control for 14 days. They were then fixed by using 4% PFA (Merck, Germany) for 30 min before staining with the Oil Red O (Merck, Germany) solution to detect lipid droplets, the Alcian Blue (Sigma Aldrich) solution to find the presence of proteoglycan and Alizarin Red S (Merck, Germany) solution to mark calcium deposition. The staining was observed under an inverted microscope equipped with a camera (Olympus Corporation, Tokyo, Japan).

To perform quantitative analysis, ADMSCs were cultured in the differentiation media for 14 days. Cells of the same passage were used as the undifferentiated control. Total RNA was extracted using RNeasy Mini Kit (Qiagen, Germany) according to the manufacturer’s instruction. cDNA synthesis was carried out using the SuperScript™ IV First-Strand Synthesis System (Invitrogen/Thermo Fisher Scientific, USA), and qPCR was performed using the 7500 Fast Real-Time PCR System and PerfeCTa SYBR Green SuperMix according to the manufacturer’s instructions. The expression of the following genes was analyzed: *PPARγ* and *Leptin* for adipogenesis, *ALP* and *PTH-R* for osteogenesis, *SOX9* for chondrogenesis and *GAPDH* for the reference gene. Primer sequences are presented in the supplementary information.

### Senescence Assay

ADMSCs were seeded on six-well plates and cultured in the PS or SM media to obtain passage 3 and passage 7 cultures. Cell senescence was analyzed using Senescence Cells Histochemical Staining Kit (Sigma Aldrich, USA) following the manufacturer’s instruction as previously described [37]. Briefly, cells were fixed and stained with Staining Mixture containing X-gal substrate overnight after reaching 70–80% confluence. Senescent cells with β-galactosidase activity were stained blue. The total number of cells was examined using DAPI (Abcam, UK) counting. Cell images were observed and captured by a IX73 microscope and cellSens software (Olympus, Japan). Cell counting was performed using Image J software (version 1.46r). The percentage of senescent cells was calculated by dividing the total number of DAPI-stained cells by the number of blue-stained cells.

### Karyotype

To evaluate the karyotype of ADMSCs after prolonged culture in vitro, passage 3 and 7 cells were checked for karyotype as previously described [26]. When the cells reached 80% confluence, they were incubated in KaryoMAX® Colcemid™ 10 µl/ ml (Gibco, USA), diluted to 1:1000, at 37 °C in a 5% CO2 incubator for 30 min. Cells were detached from the flask by incubating in 2 ml of Trypsin EDTA 0.25% (Gibco, USA) at 37 °C within 1 to 2 min and then in a pre-warmed hypotonic solution (KCl 0.56%) for 30 min. Cells were washed three times with Carnoy’s solution (3:1 v/v absolute ethanol: glacial acetic acid) before being dropped on 3–4 clean slides to spread metaphase. The slides were incubated at 60 °C overnight and stained the day after using G-banding technique. Metaphase was captured by MetaSystems, Carl Zeizz and analyzed by Ikaros software. Twelve to twenty metaphase spreads were analyzed for each sample by an experienced technician and validated by a medical genetic scientist. Karyogram is 400 band according to ISCN (An International System for Human Cytogenomic Nomenclature) 2016 standard [38].

### Statistical Analysis

All experiments in this study were performed in triplicates if not otherwise indicated. Data are present as mean ± SD if not otherwise indicated. Student t-test was used for a normally distributed population, while Mann–Whitney U test was employed in nonparametric distribution. A *p*-value below 0.05 was considered statistically significant. Differences among multiple groups were statistically analyzed using one‐way ANOVA and Tukey's multiple comparisons test. Univariate analyses were conducted using Spearman’s rank correlation and Kruskal–Wallis test. Statistical analyses were performed using the R software version 3.6.1 by a statistician [39]. Plots were prepared using the software GraphPad Prism 5.0 (GraphPad Software, La Jolla, CA).

## Results

### Increased Expression of Negative Markers in ADMSC Cultures

**Fig. 1:**
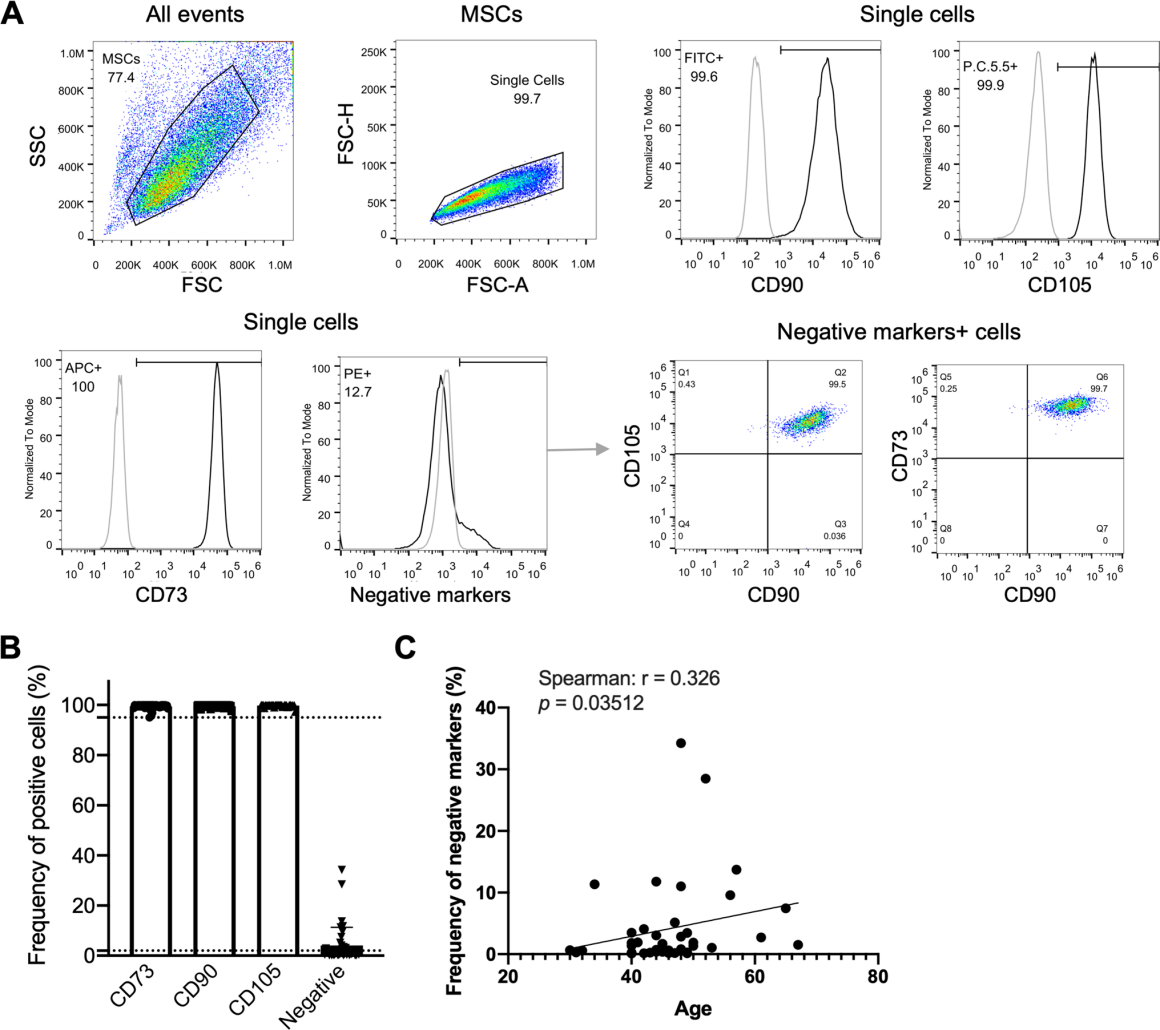
Surface marker expression in PS-cultured ADMSC samples. **(A)** A representative example of flow cytometry analysis for the expression of CD90, CD105, CD73, and the negative marker cocktail (CD34, CD45, CD11b, CD19 and HLA-DR) of an ADMSC sample cultured in PS medium. A fraction of the cells expressed negative markers. They were positive for the MSC markers CD90, CD105, and CD73. **(B)** Analysis of ADMSC samples in PS cultures (n = 42) showing that these cells displayed high levels of CD90, CD105, and CD73 and heterogenous frequencies of negative markers positive cells. The flow cytometric analysis was performed in passage 2. **(C)** The frequency of negative marker expressing cells correlates with the age of donors. Abbreviations: adipose derived mesenchymal stem/stromal cells (ADMSCs), PowerStem MSC1 media (PS)

The marker expression of ADMSCs isolated from 42 donors using the PS medium was analyzed for the MSC positive markers CD73, CD90 and CD105 and the negative or moderately expressed markers CD11b, CD19, CD34, CD45, and HLA-DR according to the ISCT’s recommendations. The samples highly expressed the markers CD73, CD90, and CD105 with more than 95% positivity (Fig. 1A and B). There are samples showing increased levels of the negative marker cocktail including CD34, CD45, CD11b, CD19, and HLA-DR (Fig. 1A). The mean values of negative marker expressing cells were 4.10% (from 0.10% to 34.24%) (Fig. 1B). Among them, 15 of the analyzed 42 samples showed more than 2% positivity for negative markers, which was the cut-off value as suggested by the ISCT. These negative marker-positive cells co-expressed CD73, CD90, and CD105 (Fig. 1A). It indicates that they represented a population of ADMSCs. Thus, our data suggest that PS medium might induce the expression of the negative markers in ADMSCs.

Next, we analyzed whether the increased expression of negative markers depends on patient characteristics such as age, gender, body mass index (BMI), and due our choice of patient cohort, of which there were 11 healthy donor and 31 patients with sexual functional deficiency. Suppl. Table 1 depicts the patient characteristics. We observed that elderly donors tended to have more negative marker-expressing cells (p = 0.035) (Fig. 1C). The other factors did not show any correlation (Suppl. Figure 2).

### Elevated Expression of the Negative Markers in PS-Cultured ADMSCs caused by HLA-DR

**Fig. 2:**
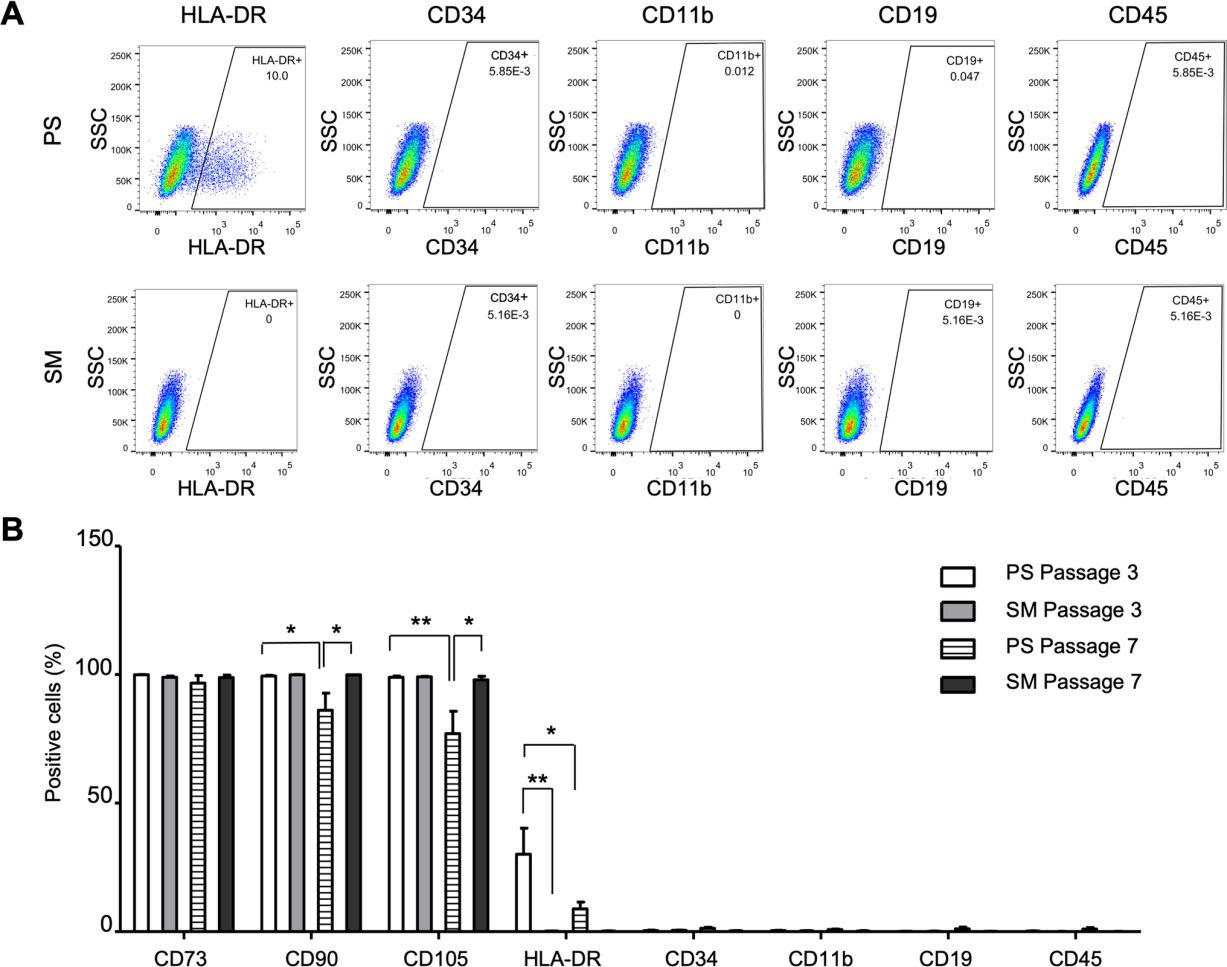
Increased HLA-DR expression in PS-cultured ADMSCs compared to their counterparts in SM. **(A)** A representative ADMSC sample cultured in PS and SM media and analyzed for their expression of individual negative markers using flow cytometry. **(B)** Differential expression of surface markers in the PS and SM cultures (n = 7). The cells in PS expressed increasingly HLA-DR compared to those in SM. In addition, the PS-cultured cells in passage 7 showed a decreased expression of CD90 and CD105. Levels of significance: *, p < 0.05. **, p < 0.01. Abbreviations: adipose derived mesenchymal stem/stromal cells (ADMSCs), PowerStem MSC1 media (PS), StemMACS MSC Expansion Media (SM)

To investigate which negative markers are increasingly expressed in the PS-cultured ADMSCs, we quantified their expression individually by flow cytometry (n = 7). For comparison, we analyzed cells derived from the same donors that were cultured in the xeno-free and serum-free SM (Fig. 2A and suppl. Figure 3). ADMSC-expanded in PS upregulated HLA-DR expression in passage 3 (mean ± SD: 27.1 ± 26.2%) and passage 7 (mean ± SD: 6.63 ± 6.59%) (Fig. 2B). In contrast, HLA-DR marker was expressed lower in ADMSC-expanded in SM, whose frequency of HLA-DR expressing cells were 0.07 ± 0.09% in passage 3 (PS versus SM: *p* = 0.003) and 0.03 ± 0.04% in passage 7 (*p* = 0.66), respectively. Although ADMSCs in the PS medium remarkably increased their HLA-DR expression in passage 3, it was decreased in the higher passage (*p* = 0.04). The other negative markers were absent in both cultures. Furthermore, ADMSCs grown in PS showed lower expression levels of CD90 and CD105 in passage 7 compared to passage 3 (*p* = 0.04 and *p* = 0.009, respectively) as well as those cultured in SM (*p* = 0.03 and *p* = 0.01, respectively) (Fig. 2B).

### The Effect of Culture Media on Cell Growth of ADMSCs

**Fig. 3:**
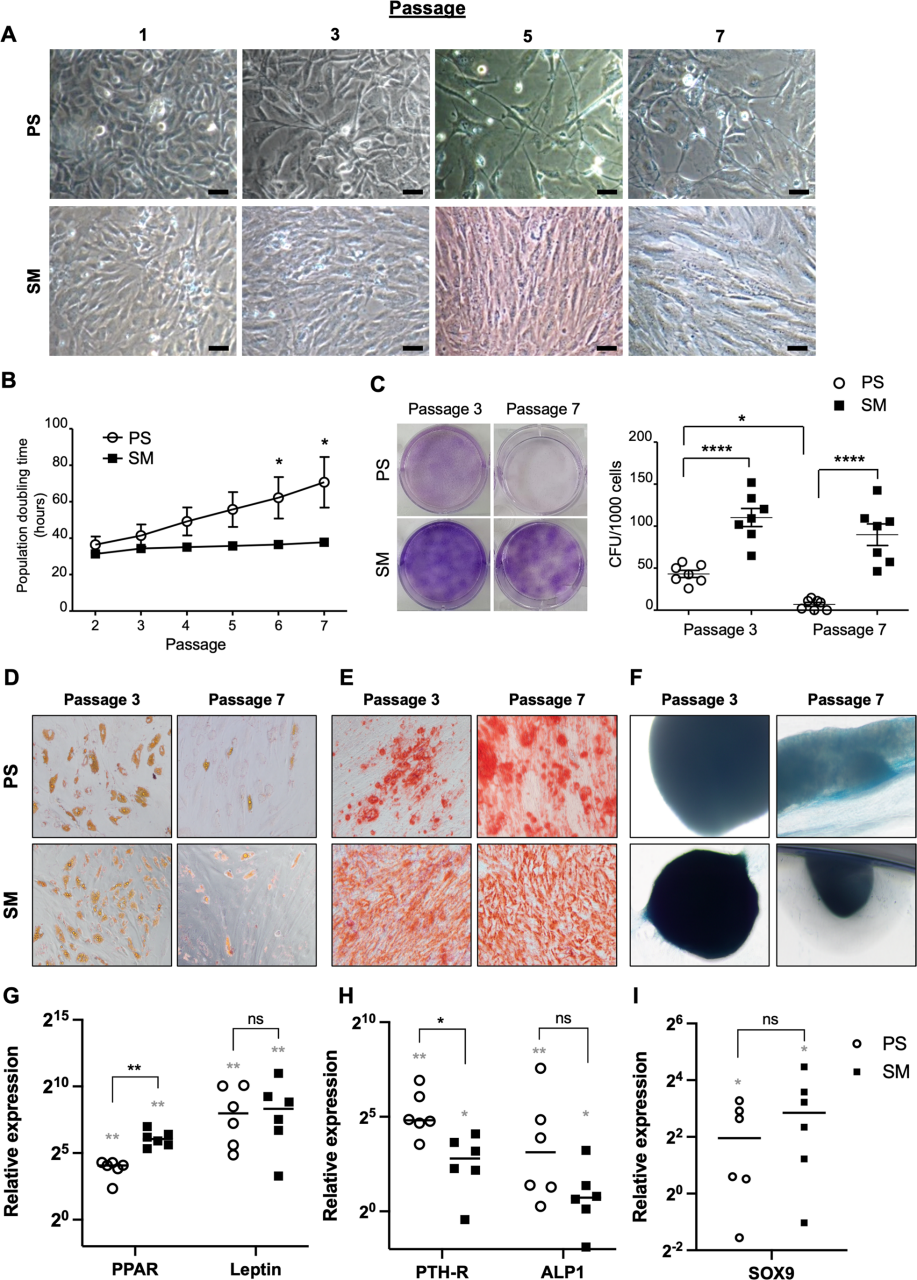
Morphology, cell growth, and differentiation of ADMSCs cultured in the PS and SM media. **(A)** Representative cell morphologies of ADMSCs cultured in the tested media from passage 1 to passage 7 (n = 7). **(B)** Population doubling time was calculated for each passage of these cells. **(C)** Colony-forming potential of ADMSCs in the media of interest. **(D-F)** ADMSCs were differentiated into adipogeneic (D), osteogeneic (E), and chondrogeneic lineages (F) and stained with Oil Red O, Alizarin Red S, and Alcian Blue, respectively. **(G-I)** Quantitative gene expression analysis was performed for adipogenic markers: *PPARγ* and *Leptin* (G)*,* osteogenic markers: *PTH-R* and *ALP* (H), and chondrogenic marker: *SOX9* (n = 6) (I)*.* Gray stars indicate the statistical significance comparing the differentiated cells with their undifferentiated controls. Black stars represent the statistic test between PS and SM cultures. Scale bars represent 100 µm. Levels of significance: *, p < 0.05. **, p < 0.01. ****, p < 0.0001. Abbreviations: adipose derived mesenchymal stem/stromal cells (ADMSCs), PowerStem MSC1 media (PS), StemMACS MSC Expansion Media (SM)

To determine the effect of media on the cell growth, ADMSCs were cultured up to passage 7 (n = 7). ADMSCs expanded in SM were small, spindle-shaped and could reach confluent during the analyzed period. In contrast, ADMSCs expanded in PS also showed small size and spindle-shaped cells in early passages but became bigger and failed to reach confluent in higher passages (Fig. 3A). Accordingly, cell proliferation rate in PS was lower than that of SM in passages 6 and 7 (*p* = 0.04 in both passages) (Fig. 3B). Furthermore, the frequencies of CFUs of the cells in PS were 4.31 ± 1.13% in passage 3 and 0.69 ± 0.61% in passage 7. They were significantly lower than the ones in SM, which were 11.03 ± 2.85% in passage 3 (*p* < 0.001) and 9.00 ± 3.40% in passage 7 (*p* < 0.001). While the CFUs of PS-expanded ADMSCs decreased in the later passage (*p* = 0.03), the colony numbers of SM-cultured cells remained high in both passages (*p* = 0.37) (Fig. 3C).

### Differentiation Capacity of ADMSCs

To test the multipotency of the ADMSC cultures, the cells were differentiated into adipogenic, osteogenic, and chondrogenic lineages. ADMSCs in both media were able to differentiate into the three cell lineages as indicated by positive staining of Oil Red O (Fig. 3D), Alizarin Red S (Fig. 3E), and Alcian Blue (Fig. 3F). Accordingly, expression of differentiation markers including *PPARγ* and *Leptin* for adipogenesis (Fig. 3G), *PTH-R* and *ALP* for osteogenesis (Fig. 3H), and *SOX9* for chondrogenesis (Fig. 3I) were increased in the differentiated samples compared to their control counterparts. The expression level of *PPARγ* was lower in the PS cultures (*p* = 0.002), while *PTH-R* was expressed higher in this condition compared to the cells in the SM media (*p* = 0.009). There were no differences in the expression of the other analyzed genes.

### Cell Viability, Proliferation, and Senescence of ADMSCs

**Fig. 4:**
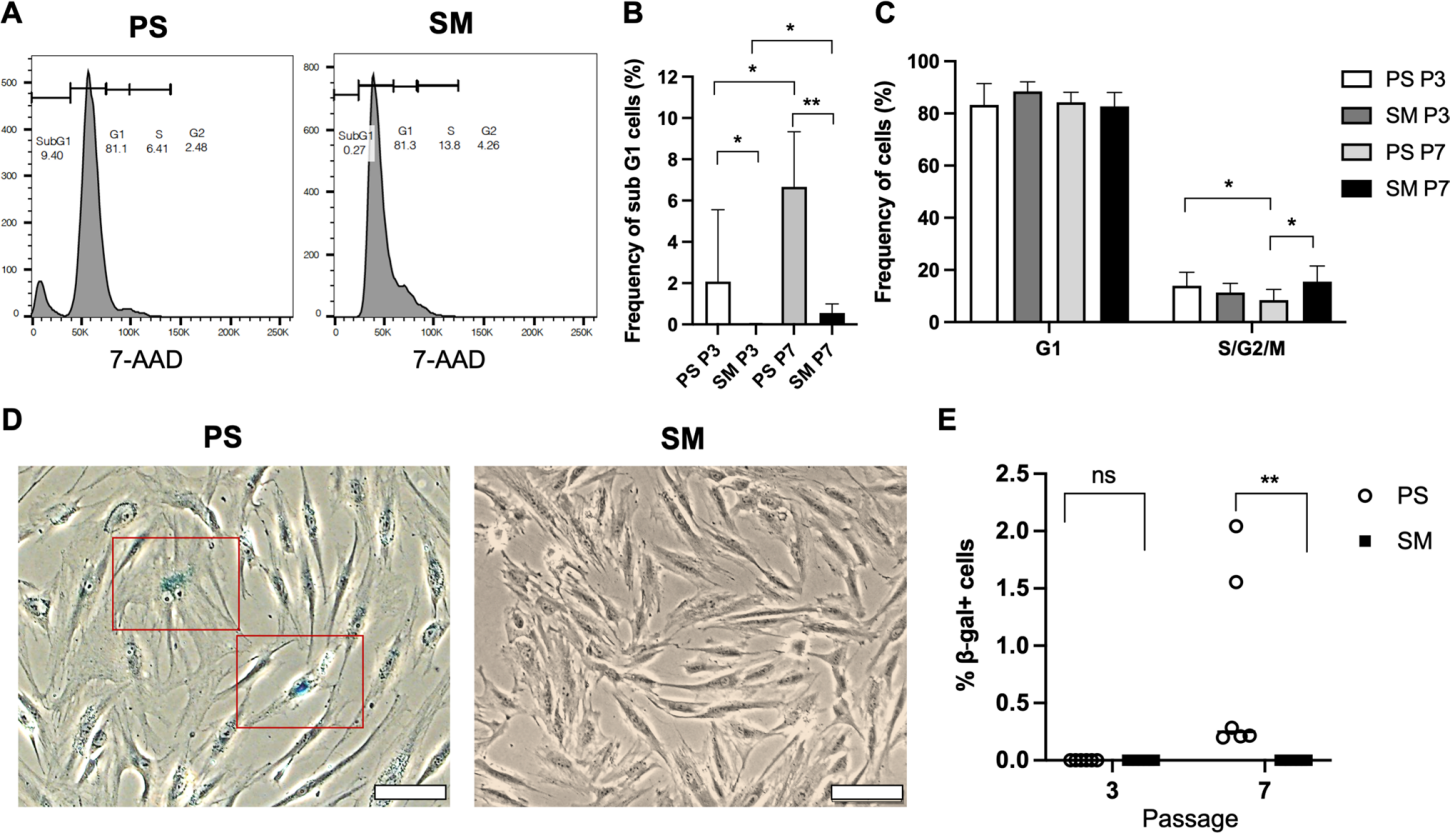
Cell cycle and senescence analysis of ADMSCs cultures. **(A)** Representative cell cycle profiles of PS and SM cultures in passage 7. Cells on sub G1, G1, S, and G2/M phases are gated based on their DNA content. **(B)** Frequencies of sub G1 cells, which represent the apoptotic population, and **(C)** frequencies of G1 and S/G2/M subsets in the PS and SM cultures in the passages 3 and 7. **(D)** In the senescence assay, senescent cells were stained in blue with b-galactosidase substrate as observed in a representative PS sample in passage 7. They were absent in the SM culture in the same passage. **(E)** No passage 3 cells were positive for b-galactosidase activity in both culture conditions. In the higher passage, PS cultures displayed an increased frequency of senescent cells compared to those in SM. Scale bars represent 100 µm. Abbreviations: adipose derived mesenchymal stem/stromal cells (ADMSCs), passage (P), PowerStem MSC1 media (PS), StemMACS MSC Expansion Media (SM)

Cell viability of ADMSCs in PS and SM cultures were analyzed using trypan blue and 7-AAD. There were no differences in the numbers of dead cells in both passages 3 and 7 (Suppl. Figure 4). Furthermore, the cells were fixed and stained with 7-AAD to analyze their cell cycle (Fig. 4A). Apoptotic cells, which have reduced DNA content compared to living cells, were observed in the sub G1 phase. Their frequencies were increased in the PS compared to the SM cultures in both passages 3 and 7 (Fig. 4B). Accordingly, the proportion of proliferating cells in the S/G2/M phases decreased in the PS cultures in passage 7 compared to their counterparts in SM as well as the PS cells in passage 3 (Fig. 4C). The results indicated a reduced proliferation capacity of the PS cells in a higher passage.

Therefore, senescence analysis was performed using the b-galactosidase assay (Fig. 4D). Both cultures in passage 3 were free of b-galactosidase positive cells. In passage 7, cells cultured in PS showed a higher frequency of senescent cells compared to those in SM (Fig. 4E). Thus, our results of cell cycle and cellular senescence indicated that the later media was able to maintain ADMSCs longer in a proliferative state.

### Changes of Karyotype of ADMSCs during *in vitro* Culture

**Fig. 5:**
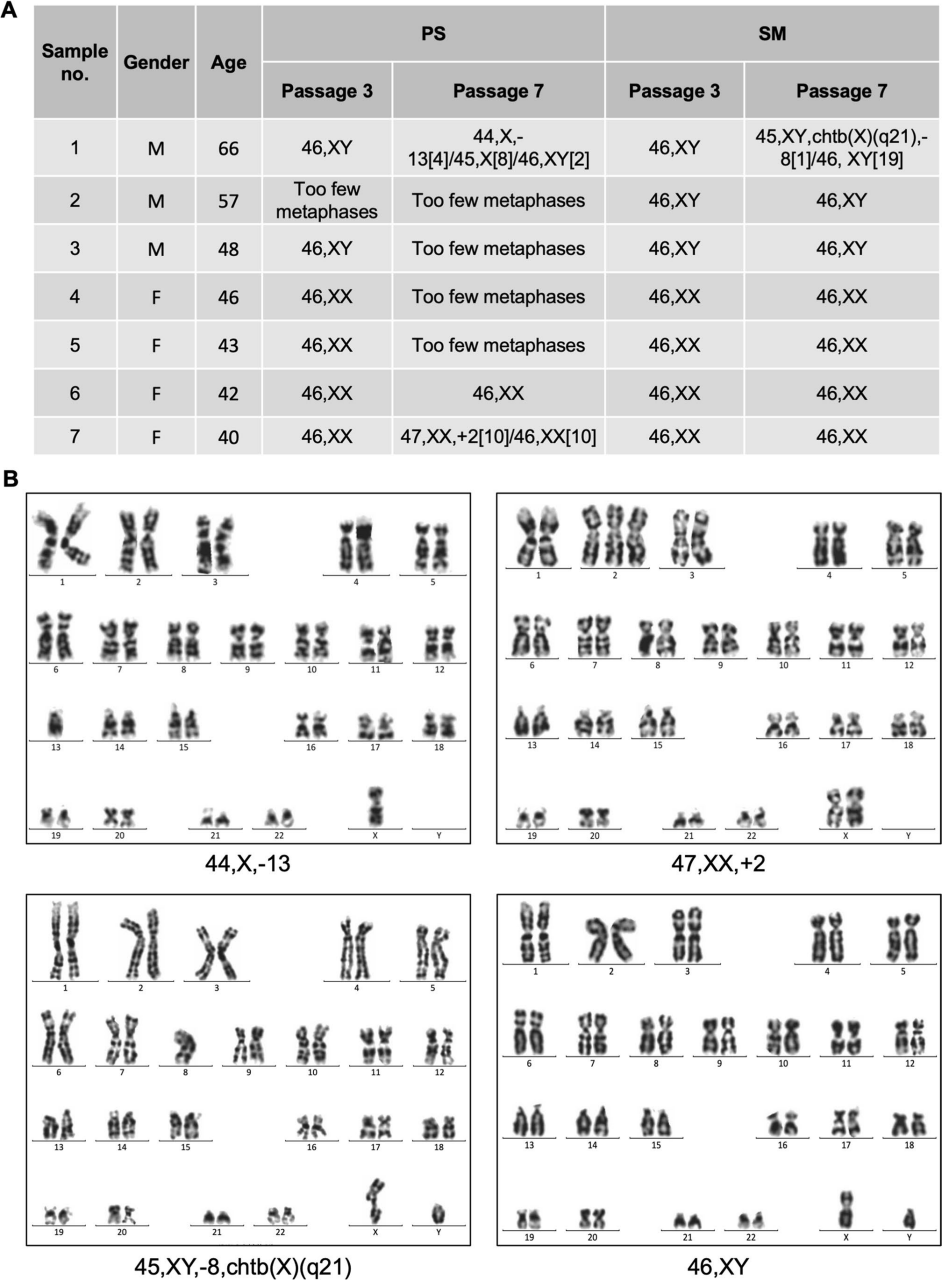
Karyotype analysis of ADMSCs cultured in the PS and SM media. **(A)** Karyotypes of seven analyzed ADMSC samples in the passages 3 and 7 that was sorted by the age of the donors. **(B)** Representative examples of abnormal and normal karyotypes. Abbreviations: adipose derived mesenchymal stem/stromal cells (ADMSCs), PowerStem MSC1 media (PS), StemMACS MSC Expansion Media (SM)

To investigate whether the changes in cell growth rates, morphological and immunophenotypic characteristics as well as senescence were associated with genome instability, we performed karyotyping of ADMSCs (Fig. 5A and B). MSCs of sample 1 displayed a normal karyotype in passage 3 in both media; however, four and eight of 13 analyzed cells expanded in PS at passage 7 displayed deletions of chromosome 13 and chromosome Y, respectively. The cells cultured in SM showed a deletion of chromosome 8 and chtb(X)(q21) in one of 20 analyzed cells, which was likely a random loss of chromosomes due to technical preparation of the slide. The sample 7 had an addition of chromosome 2 in half of the PS-expanded cells in passage 7, while the other cultures derived from the same sample showed a normal karyotype. In four of the seven analyzed samples, there were too few metaphases in the PS culture in passage 7. It was correlated with their declined proliferation compared to the SM cultivated counterparts as shown in Fig. 3B. Interestingly, mutations occurred in both samples only in passage 7 but not in passage 3 suggesting that these chromosomal abnormalities were induced during the ex vivo expansion.

## Discussion

The xeno-free and serum-free medium PS has been used to manufacture ADMSCs and umbilical cord-derived MSCs in some clinical trials [34, 40, 41]. However, there is only limited data reporting the immunophenotype of MSCs in this medium so far. Recently, our group observed that ADMSCs cultured in PS exhibited evaluated levels of negative markers [35]. For the first time, we demonstrated that PS induced HLA-DR upregulation in these cells. It is a concern regarding the minimal criteria for MSCs established by the ISCT [27, 28]. An increased HLA-DR expression was also observed in GMP manufactured bone marrow-derived MSCs (BMMSCs) using either human sera or platelet lysate supplemented media [42]. The authors reported that the HLA-DR expression was unpredictable and dynamic over time in those cases [43]. Our data suggested that donor age was correlated with the increased signal of HLA-DR. In line with this observation, PS only induced its overexpression in adult MSCs, but not in those derived from prenatal tissues such as the umbilical cord [35].

Like PS, SM was able to support the isolation and expansion of ADMSCs [35]. Therefore, we performed a comparative study of these two xeno-free and serum-free culture media using the same samples and a common protocol. Our data demonstrated that both media were capable to differentiate ADMSCs into adipogenic, osteogenic, and chondrogenic lineages. However, SM supported cell growth and prevented ADMSCs from senescence better than PS. Interestingly, only PS medium triggered the expression of HLA-DR and displayed lower expression of CD90 and CD105 in passage 7, while SM medium showed no changes in marker profile during culture time. This indicates that PS itself should be the reason for the observed changes in the cells. Indeed, several factors in culture media can directly influence the MSC phenotype. The expression of HLA-DR can be induced in vitro by incubation of MSCs with IFN-γ or other pro-inflammatory cytokines [42–44] and basic fibroblast growth factor (bFGF) [45, 46]. In addition, CD105 level reduced in BMMSCs after pro-longed culture under a serum-free condition or during chondrogenic differentiation in the presence of transforming growth factor beta 3 [47, 48]. However, it remains elusive which factors in the PS medium caused the altered characteristics of ADMSCs.

The consequence of an HLA-DR upregulation on the bioactivity of MSCs remains controversial. Previous reports suggested that the HLA-DR overexpression on BMMSCs did not change their identity and potency [42, 43]. In contrast, our data indicated that in addition to HLA-DR induction, ADMSCs changed their karyotypes and earlier senescence upon a prolonged culture in the PS medium. For comparison, the SM medium showed a more stable karyotype and higher proliferation capacity under the same condition.

Furthermore, HLA-DR-expressing ADMSCs in the PS culture might increase the risk of alloimmune response as previously discussed for allogenic HLA-DR^+^ MSCs [49]. Several animal studies have shown that MSCs triggered immune reactions and resulted in a rejection of the administrated cells in HLA-mismatched mice [29, 50, 51], pigs [30], horses [31, 32], and monkeys [52]. On the other hand, INF-γ significantly enhanced the immunosuppressive effects of primed MSCs cells, although it induced an upregulation of HLA-ABC and HLA-DR [53–55]. Thus, the impact of HLA-DR expression on clinical use needs to be further investigated.

## Conclusion

The xeno-free and serum-free PS medium was unsuitable for the expansion of ADMSCs, because it induced HLA-DR expression and led to decreased cell growth and proliferation as well as abnormal karyotypes in contrast to the SM medium. In addition, we provided evidence for a correlation between the increased expression of negative markers and the donor’s age in MSCs. These findings suggest the importance of culture media for the features of MSC-based products and emphasize the necessity of intensive quality control of these cells for a successful translation from bench to bedside.

## Supplementary Information

Below is the link to the electronic supplementary material.

Supplementary file1 (DOCX 15.1 MB)

## Data Availability

The datasets used and/or analyzed during the current study are available from the corresponding author on reasonable request.

## References

[1] Zuk, P. A., Zhu, M., Ashjian, P., De Ugarte, D. A., Huang, J. I., Mizuno, H., …, & Hedrick, M. H. (2002). Human adipose tissue is a source of multipotent stem cells. *Molecular Biology of the Cell*, *13*(12), 4279–4295. 10.1091/mbc.e02-02-0105. 10.1091/mbc.E02-02-0105 PMC138633 12475952

[2] Ashjian, P. H., Elbarbary, A. S., Edmonds, B., DeUgarte, D., Zhu, M., Zuk, P. A., …, & Hedrick, M. H. (2003). In Vitro differentiation of human processed lipoaspirate cells into early neural progenitors: *Plastic and Reconstructive Surgery*, *111*(6), 1922–1931. 10.1097/01.PRS.0000055043.62589.05. 10.1097/01.PRS.0000055043.62589.05 12711954

[3] Safford KM, Hicok KC, Safford SD, Halvorsen Y-DC, Wilkison WO, Gimble JM, Rice HE (2002). Neurogenic differentiation of murine and human adipose-derived stromal cells. Biochemical and Biophysical Research Communications.

[4] Seo MJ, Suh SY, Bae YC, Jung JS (2005). Differentiation of human adipose stromal cells into hepatic lineage in vitro and in vivo. Biochemical and Biophysical Research Communications.

[5] Schmelzer E, McKeel DT, Gerlach JC (2019). Characterization of human mesenchymal stem cells from different tissues and their membrane encasement for prospective transplantation therapies. BioMed Research International.

[6] Bateman ME, Strong AL, Gimble JM, Bunnell BA (2018). Concise review: Using fat to fight disease: A systematic review of nonhomologous adipose-derived stromal/stem cell therapies: Using fat to fight disease. STEM CELLS.

[7] Galipeau J, Sensébé L (2018). Mesenchymal stromal cells: Clinical challenges and therapeutic opportunities. Cell Stem Cell.

[8] Kabat M, Bobkov I, Kumar S, Grumet M (2020). Trends in mesenchymal stem cell clinical trials 2004–2018: Is efficacy optimal in a narrow dose range?. STEM CELLS Translational Medicine.

[9] Lensch M, Muise A, White L, Badowski M, Harris D (2018). Comparison of synthetic media designed for expansion of adipose-derived mesenchymal stromal cells. Biomedicines.

[10] Panés, J., García-Olmo, D., Van Assche, G., Colombel, J. F., Reinisch, W., Baumgart, D. C., …, & Danese, S. (2016). Expanded allogeneic adipose-derived mesenchymal stem cells (Cx601) for complex perianal fistulas in Crohn’s disease: a phase 3 randomised, double-blind controlled trial. *The Lancet*, *388*(10051), 1281–1290. 10.1016/S0140-6736(16)31203-X. 10.1016/S0140-6736(16)31203-X 27477896

[11] Lee W, Kim HJ, Kim K, Kim GB, Jin W (2019). Intra-articular injection of autologous adipose tissue-derived mesenchymal stem cells for the treatment of knee Osteoarthritis: A phase IIb, randomized, placebo-controlled clinical trial. STEM CELLS Translational Medicine.

[12] Lu, L., Dai, C., Zhang, Z., Du, H., Li, S., Ye, P., …, & Bao, C. (2019). Treatment of knee osteoarthritis with intra-articular injection of autologous adipose-derived mesenchymal progenitor cells: a prospective, randomized, double-blind, active-controlled, phase IIb clinical trial. *Stem Cell Research & Therapy*, *10*(1), 143. 10.1186/s13287-019-1248-3. 10.1186/s13287-019-1248-3 PMC6528322 31113476

[13] Freitag, J., Bates, D., Wickham, J., Shah, K., Huguenin, L., Tenen, A., …, & Boyd, R. (2019). Adipose-derived mesenchymal stem cell therapy in the treatment of knee osteoarthritis: a randomized controlled trial. *Regenerative Medicine*, *14*(3), 213–230. 10.2217/rme-2018-0161. 10.2217/rme-2018-0161 30762487

[14] DiGirolamo CM, Stokes D, Colter D, Phinney DG, Class R, Prockop DJ (1999). Propagation and senescence of human marrow stromal cells in culture: A simple colony-forming assay identifies samples with the greatest potential to propagate and differentiate: Human Marrow Stromal Cells in Culture. British Journal of Haematology.

[15] Sekiya I, Larson BL, Smith JR, Pochampally R, Cui J, Prockop DJ (2002). Expansion of human adult stem cells from bone marrow stroma: Conditions that maximize the yields of early progenitors and evaluate their quality. Stem Cells.

[16] Riis S, Nielsen FM, Pennisi CP, Zachar V, Fink T (2016). Comparative analysis of media and supplements on initiation and expansion of adipose-derived stem cells: Effect of media on ASCs. STEM CELLS Translational Medicine.

[17] Bakopoulou, A., Apatzidou, D., Aggelidou, E., Gousopoulou, E., Leyhausen, G., Volk, J., …, & Geurtsen, W. (2017). Isolation and prolonged expansion of oral mesenchymal stem cells under clinical-grade, GMP-compliant conditions differentially affects “stemness” properties. *Stem Cell Research & Therapy*, *8*(1), 247. 10.1186/s13287-017-0705-0. 10.1186/s13287-017-0705-0 PMC5667471 29096714

[18] Crisostomo, P. R., Wang, M., Wairiuko, G. M., Morrell, E. D., Terrell, A. M., Seshadri, P., …, & Meldrum, D. R. (2006). High passage number of stem cells adversely affects stem cell activation and myocardial protection: *Shock*, *26*(6), 575–580. 10.1097/01.shk.0000235087.45798.93. 10.1097/01.shk.0000235087.45798.93 17117132

[19] Nikitina, V., Astrelina, T., Nugis, V., Ostashkin, A., Karaseva, T., Dobrovolskaya, E., …, & Samoilov, A. (2018). Clonal chromosomal and genomic instability during human multipotent mesenchymal stromal cells long-term culture. *PLoS One*, *13*(2), e0192445. 10.1371/journal.pone.0192445. 10.1371/journal.pone.0192445 PMC5809118 29432491

[20] Stultz BG, McGinnis K, Thompson EE, Lo Surdo JL, Bauer SR, Hursh DA (2016). Chromosomal stability of mesenchymal stromal cells during in vitro culture. Cytotherapy.

[21] Pan Q, Fouraschen SM, de Ruiter PE, Dinjens WN, Kwekkeboom J, Tilanus HW, van der Laan LJ (2014). Detection of spontaneous tumorigenic transformation during culture expansion of human mesenchymal stromal cells. Experimental Biology and Medicine.

[22] Marędziak M, Marycz K, Tomaszewski KA, Kornicka K, Henry BM (2016). The influence of aging on the regenerative potential of human adipose derived mesenchymal stem cells. Stem Cells International.

[23] Kornicka K, Marycz K, Tomaszewski KA, Marędziak M, Śmieszek A (2015). The effect of age on osteogenic and adipogenic differentiation potential of Human Adipose Derived Stromal Stem Cells (hASCs) and the impact of stress factors in the course of the differentiation process. Oxidative Medicine and Cellular Longevity.

[24] Yang Y-HK (2018). Aging of mesenchymal stem cells: Implication in regenerative medicine. Regenerative Therapy.

[25] Kornicka K, Houston J, Marycz K (2018). Dysfunction of mesenchymal stem cells isolated from metabolic syndrome and type 2 diabetic patients as result of oxidative stress and autophagy may limit their potential therapeutic use. Stem Cell Reviews and Reports.

[26] Nguyen, L. T., Hoang, D. M., Nguyen, K. T., Bui, D. M., Nguyen, H. T., Le, H. T. A., …, & Bui, A. V. (2021). Type 2 diabetes mellitus duration and obesity alter the efficacy of autologously transplanted bone marrow‐derived mesenchymal stem/stromal cells. *STEM CELLS Translational Medicine*, sctm.20–0506. 10.1002/sctm.20-0506. 10.1002/sctm.20-0506 PMC8380443 34080789

[27] Dominici, M., Le Blanc, K., Mueller, I., Slaper-Cortenbach, I., Marini, F. C., Krause, D. S., …, & Horwitz, E. M. (2006). Minimal criteria for defining multipotent mesenchymal stromal cells. The International Society for Cellular Therapy position statement. *Cytotherapy*, *8*(4), 315–317. 10.1080/14653240600855905. 10.1080/14653240600855905 16923606

[28] Bourin, P., Bunnell, B. A., Casteilla, L., Dominici, M., Katz, A. J., March, K. L., …, & Gimble, J. M. (2013). Stromal cells from the adipose tissue-derived stromal vascular fraction and culture expanded adipose tissue-derived stromal/stem cells: a joint statement of the International Federation for Adipose Therapeutics and Science (IFATS) and the International Society for Cellular Therapy (ISCT). *Cytotherapy*, *15*(6), 641–648. 10.1016/j.jcyt.2013.02.006. 10.1016/j.jcyt.2013.02.006 PMC3979435 23570660

[29] Eliopoulos N, Stagg J, Lejeune L, Pommey S, Galipeau J (2005). Allogeneic marrow stromal cells are immune rejected by MHC class I– and class II–mismatched recipient mice. Blood.

[30] Poncelet AJ, Vercruysse J, Saliez A, Gianello P (2007). Although pig allogeneic mesenchymal stem cells are not immunogenic In Vitro intracardiac injection elicits an immune response In Vivo. Transplantation.

[31] Owens SD, Kol A, Walker NJ, Borjesson DL (2016). Allogeneic mesenchymal stem cell treatment induces specific alloantibodies in horses. Stem Cells International.

[32] Pezzanite LM, Fortier LA, Antczak DF, Cassano JM, Brosnahan MM, Miller D, Schnabel LV (2015). Equine allogeneic bone marrow-derived mesenchymal stromal cells elicit antibody responses in vivo. Stem Cell Research & Therapy.

[33] Chang S-H, Kim HJ, Park C-G (2020). Allogeneic ADSCs induce the production of alloreactive memory-CD8 T cells through HLA-ABC antigens. Cells.

[34] Nguyen Thanh, L., Dam, P. T. M., Nguyen, H.-P., Nguyen, T.-S. T., To, H. M., Nguyen, H. B., …, & Hoang, D. M. (2021). Can autologous adipose-derived mesenchymal stem cell transplantation improve sexual function in people with sexual functional deficiency? *Stem Cell Reviews and Reports*. 10.1007/s12015-021-10196-w. 10.1007/s12015-021-10196-w 34129158

[35] Hoang, V. T., Trinh, Q.-M., Phuong, D. T. M., Bui, H. T. H., Hang, L. M., Ngan, N. T. H., …, & Hoang, D. M. (2020). Standardized xeno- and serum-free culture platform enables large-scale expansion of high-quality mesenchymal stem/stromal cells from perinatal and adult tissue sources. *Cytotherapy*, S1465324920308562. 10.1016/j.jcyt.2020.09.004. 10.1016/j.jcyt.2020.09.004 33097415

[36] Hoang, V. T., Verma, D., Godavarthy, P. S., Llavona, P., Steiner, M., Gerlach, K., …, & Krause, D. S. (2019). The transcriptional regulator FUBP1 influences disease outcome in murine and human myeloid leukemia. *Leukemia*, *33*(7), 1700–1712. 10.1038/s41375-018-0358-8. 10.1038/s41375-018-0358-8 30635626

[37] Hoang, D. H., Nguyen, T. D., Nguyen, H.-P., Nguyen, X.-H., Do, P. T. X., Dang, V. D., …, & Than, U. T. T. (2020). Differential wound healing capacity of mesenchymal stem cell-derived exosomes originated from bone marrow, adipose tissue and umbilical cord under serum- and xeno-free condition. *Frontiers in Molecular Biosciences*, *7*, 119. 10.3389/fmolb.2020.00119. 10.3389/fmolb.2020.00119 PMC7327117 32671095

[38] McGowan-Jordan J, Simons A, Schmid M (2016). An International System for Human Cytogenomic Nomenclature (2016).

[39] R Core Team. (2013). *R: A language and environment for statistical computing.* R Foundation for Statistical Computing, Vienna, Austria. Retrieved from http://www.R-project.org/. Accessed 27 Aug 2021.

[40] Nguyen, L. T., Trieu, T. T. H., Bui, H. T. H., Hoang, V. T., Nguyen, A. T. T., Trinh, N. T. H., …, & Hoang, D. M. (2020). Allogeneic administration of human umbilical cord-derived mesenchymal stem/stromal cells for bronchopulmonary dysplasia: preliminary outcomes in four Vietnamese infants. *Journal of Translational Medicine*, *18*(1), 398. 10.1186/s12967-020-02568-6. 10.1186/s12967-020-02568-6 PMC7576694 33081796

[41] Tak YJ, Lee SY, Cho AR, Kim YS (2020). A randomized, double-blind, vehicle-controlled clinical study of hair regeneration using adipose-derived stem cell constituent extract in androgenetic alopecia. STEM CELLS Translational Medicine.

[42] Grau-Vorster, M., Rodríguez, L., Torrents-Zapata, S., Vivas, D., Codinach, M., Blanco, M., …, & Vives, J. (2019). Levels of IL-17F and IL-33 correlate with HLA-DR activation in clinical-grade human bone marrow–derived multipotent mesenchymal stromal cell expansion cultures. *Cytotherapy*, *21*(1), 32–40. 10.1016/j.jcyt.2018.09.009. 10.1016/j.jcyt.2018.09.009 30447901

[43] Grau-Vorster M, Laitinen A, Nystedt J, Vives J (2019). HLA-DR expression in clinical-grade bone marrow-derived multipotent mesenchymal stromal cells: A two-site study. Stem Cell Research & Therapy.

[44] Kim J-H, Jo CH, Kim H-R, Hwang Y (2018). Comparison of immunological characteristics of mesenchymal stem cells from the periodontal ligament, umbilical cord, and adipose tissue. Stem Cells International.

[45] Bocelli-Tyndall, C., Zajac, P., Di Maggio, N., Trella, E., Benvenuto, F., Iezzi, G., …, & Tyndall, A. (2010). Fibroblast growth factor 2 and platelet-derived growth factor, but not platelet lysate, induce proliferation-dependent, functional class II major histocompatibility complex antigen in human mesenchymal stem cells: Induction of Class II MHC Antigen in Human MSCs. *Arthritis & Rheumatism*, *62*(12), 3815–3825. 10.1002/art.27736. 10.1002/art.27736 20824797

[46] Dighe PA, Viswanathan P, Mruthunjaya AK, Seetharam RN (2013). Effect of bFGF on HLA-DR expression of human bone marrow-derived mesenchymal stem cells. Journal of Stem Cells.

[47] Mark, P., Kleinsorge, M., Gaebel, R., Lux, C. A., Toelk, A., Pittermann, E., …, & Ma, N. (2013). Human Mesenchymal Stem Cells Display Reduced Expression of CD105 after Culture in Serum-Free Medium. *Stem Cells International*, *2013*, 1–8. 10.1155/2013/698076. 10.1155/2013/698076 PMC3806428 24194767

[48] Lee HJ, Choi BH, Min B-H, Park SR (2009). Changes in surface markers of human mesenchymal stem cells during the chondrogenic differentiation and dedifferentiation processes in vitro. Arthritis & Rheumatism.

[49] Kot M, Baj-Krzyworzeka M, Szatanek R, Musiał-Wysocka A, Suda-Szczurek M, Majka M (2019). The importance of HLA assessment in “Off-the-Shelf” allogeneic mesenchymal stem cells based-therapies. International Journal of Molecular Sciences.

[50] Nauta AJ, Westerhuis G, Kruisselbrink AB, Lurvink EGA, Willemze R, Fibbe WE (2006). Donor-derived mesenchymal stem cells are immunogenic in an allogeneic host and stimulate donor graft rejection in a nonmyeloablative setting. Blood.

[51] Zangi L, Margalit R, Reich-Zeliger S, Bachar-Lustig E, Beilhack A, Negrin R, Reisner Y (2009). Direct imaging of immune rejection and memory induction by allogeneic mesenchymal stromal cells. Stem Cells.

[52] Isakova IA, Lanclos C, Bruhn J, Kuroda MJ, Baker KC, Krishnappa V, Phinney DG (2014). Allo-aeactivity of mesenchymal stem cells in Rhesus Macaques is dose and haplotype dependent and limits durable cell engraftment In Vivo. PLoS One.

[53] Kim, D. S., Jang, I. K., Lee, M. W., Ko, Y. J., Lee, D.-H., Lee, J. W., …, & Yoo, K. H. (2018). Enhanced immunosuppressive properties of human mesenchymal stem cells primed by interferon-γ. *EBioMedicine*, *28*, 261–273. 10.1016/j.ebiom.2018.01.002. 10.1016/j.ebiom.2018.01.002 PMC5898027 29366627

[54] Noronha, N. de C., Mizukami, A., Caliári-Oliveira, C., Cominal, J. G., Rocha, J. L. M., Covas, D. T., …, & Malmegrim, K. C. R. (2019). Priming approaches to improve the efficacy of mesenchymal stromal cell-based therapies. *Stem Cell Research & Therapy*, *10*(1), 131. 10.1186/s13287-019-1224-y. 10.1186/s13287-019-1224-y PMC6498654 31046833

[55] Sheng, H., Wang, Y., Jin, Y., Zhang, Q., Zhang, Y., Wang, L., …, & Li, N. (2008). A critical role of IFNγ in priming MSC-mediated suppression of T cell proliferation through up-regulation of B7-H1. *Cell Research*, *18*(8), 846–857. 10.1038/cr.2008.80. 10.1038/cr.2008.80 18607390

