# Supplementary material for: Human Adipose-Derived Mesenchymal Stromal Cells Exhibit High HLA-DR Levels and Altered Cellular Characteristics under a Xeno-free and Serum-free Condition
Source: Stem Cell Rev Rep. 2021 Sep 11;17(6):2291–303. doi: 10.1007/s12015-021-10242-7 (PMC8599375; doi:10.1007/s12015-021-10242-7)

**Supplementary information**

**Human Adipose-derived Mesenchymal Stromal Cells Exhibit High HLA-DR Levels and Altered Cellular Characteristics under a Xeno-free and Serum-free Condition**

Phuong TM Dam^1*^, Van T. Hoang^2*^, Hue Thi Hong Bui^1^, Le Minh Hang^1^, Duc M. Hoang^2^, Hoang Phuong Nguyen^2^, Lien Ha Thi^1^, Huong Tran Thi Thanh^1^, Xuan-Hung Nguyen^1^, Liem Nguyen Thanh^2#^

^1^Vinmec Institute of Applied Science and Regenerative Medicine, Vinmec Health Care System, Hanoi, Vietnam

^2^Vinmec Research Institute of Stem Cell and Gene Technology (VRISG), Vinmec Health Care System, Hanoi, Vietnam

**Suppl. Table 1: Patient characteristics**

|  | **Overall (N=42)** |
| --- | --- |
| **Sex** |  |
| Male | 18 (42.9%) |
| Female | 24 (57.1%) |
| **Weight** |  |
| Mean (SD) | 60.3 (9.76) |
| Median [Min, Max] | 60.0 [43.0, 90.0] |
| **Height** |  |
| Mean (SD) | 1.62 (0.0648) |
| Median [Min, Max] | 1.61 [1.48, 1.76] |
| **Age** |  |
| Mean (SD) | 46.2 (7.86) |
| Median [Min, Max] | 46.0 [31.0, 67.0] |
| **BMI** |  |
| Mean (SD) | 22.9 (2.41) |
| Median [Min, Max] | 23.0 [17.7, 29.1] |
| **Patient cohort** |  |
| Patients with sexual hormone deficiency | 32 (73,81%) |
| Healthy donors | 11 (26,19%) |

**Suppl. Table 2: Primer sequences**

| **Gene** | **Lineage** | **Primer** | **Primer sequence** |
| --- | --- | --- | --- |
| PPARg | Adipocyte | Forward Primer | CCCTTCACTACTGTTGACTTCTC |
| PPARg | Adipocyte | Reverse Primer | TGCAGGCTCCACTTTGATT |
| Leptin | Adipocyte | Forward Primer | CCAGGATCAATGACATTTCACAC |
| Leptin | Adipocyte | Reverse Primer | CATACTGGTGAGGATCTGTTGG |
| ALP | Osteocyte | Forward Primer | CCCGTGGCAACTCTATCTT |
| ALP | Osteocyte | Reverse Primer | AGTCCACCATGGAGACATTC |
| PTH-R | Osteocyte | Forward Primer | TGAACGGGAGGTGTTTGA |
| PTH-R | Osteocyte | Reverse Primer | GGTGCATGTGGATGTAGTTG |
| Sox9 | Chondrocyte | Forward Primer | AACAAGCCGCACGTCAA |
| Sox9 | Chondrocyte | Reverse Primer | TCTCGCTCTCGTTCAGAAGT |
| GAPDH | Housekeeping genes | Forward Primer | GGTGTGAACCATGAGAAGTATGA |
| GAPDH | Housekeeping genes | Reverse Primer | GAGTCCTTCCACGATACCAAAG |

**Figure legends:**

**Suppl. Figure 1: Study design.** The immunophenotype of ADMSC samples expanded in PS were analysed and tested for correlations with patient characteristics (n=42). Next, we cultured seven samples in two xeno-free and serum-free PS and SM media and investigated their bioactivity including: growth properties, CFU numbers, immunophenotype, differentiation ability, cell proliferation, senescence, and karyotype. Abbreviations: adipose derived mesenchymal stem/stromal cells (ADMSCs), PowerStem MSC1 media (PS), StemMACS MSC Expansion Media (SM).

**Suppl. Figure 2**: **Correlation between the expression of negative markers and patient characteristics**: **(A)** gender, **(B)** BMI, and **(C)** based on patient cohorts: healthy donors of those with sexual functional deficiency.

**Suppl. Figure 3**: **Surface marker expression of ADMSCs.** A representative example of flow cytometry analysis for the expression of CD90, CD105, CD73, and the negative marker cocktail (CD34, CD45, CD11b, CD19 and HLA-DR) of an ADMSC sample cultured in PS and SM media at **(A)** passage 3 and **(B)** passage 7. Abbreviations: adipose derived mesenchymal stem/stromal cells (ADMSCs), PowerStem MSC1 media (PS), StemMACS MSC Expansion Media (SM).

**Suppl. Figure 4: Cell viability of ADMSCs cultured in PS and SM using (A) trypan blue and (B) 7-AAD.** Abbreviations: adipose derived mesenchymal stem/stromal cells (ADMSCs), PowerStem MSC1 media (PS), StemMACS MSC Expansion Media (SM).


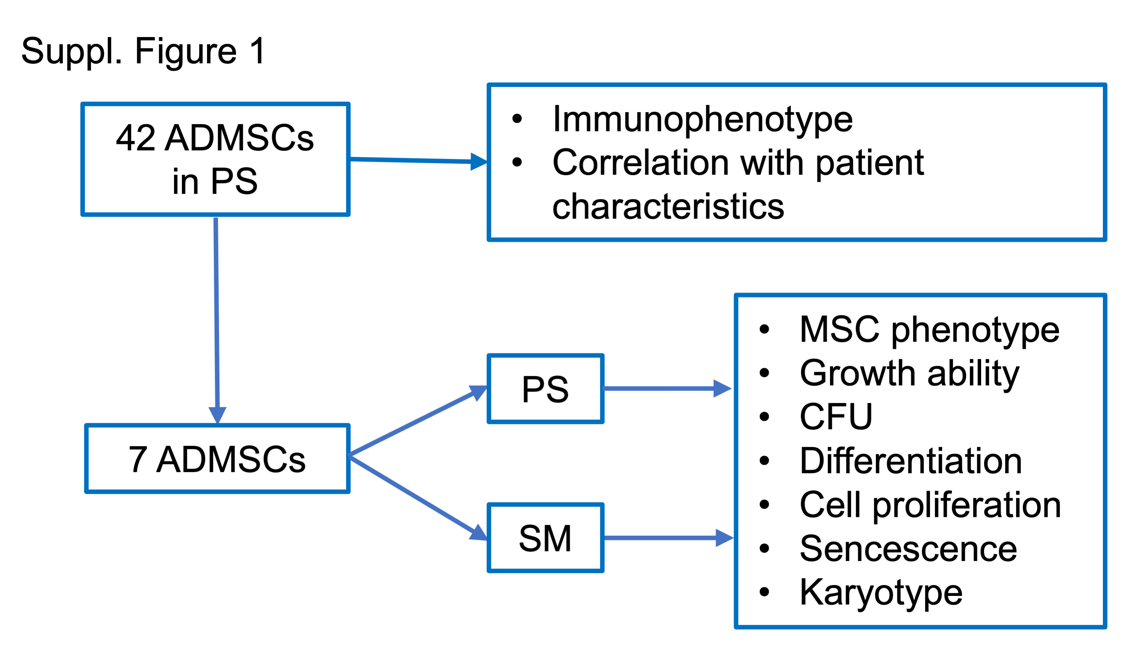


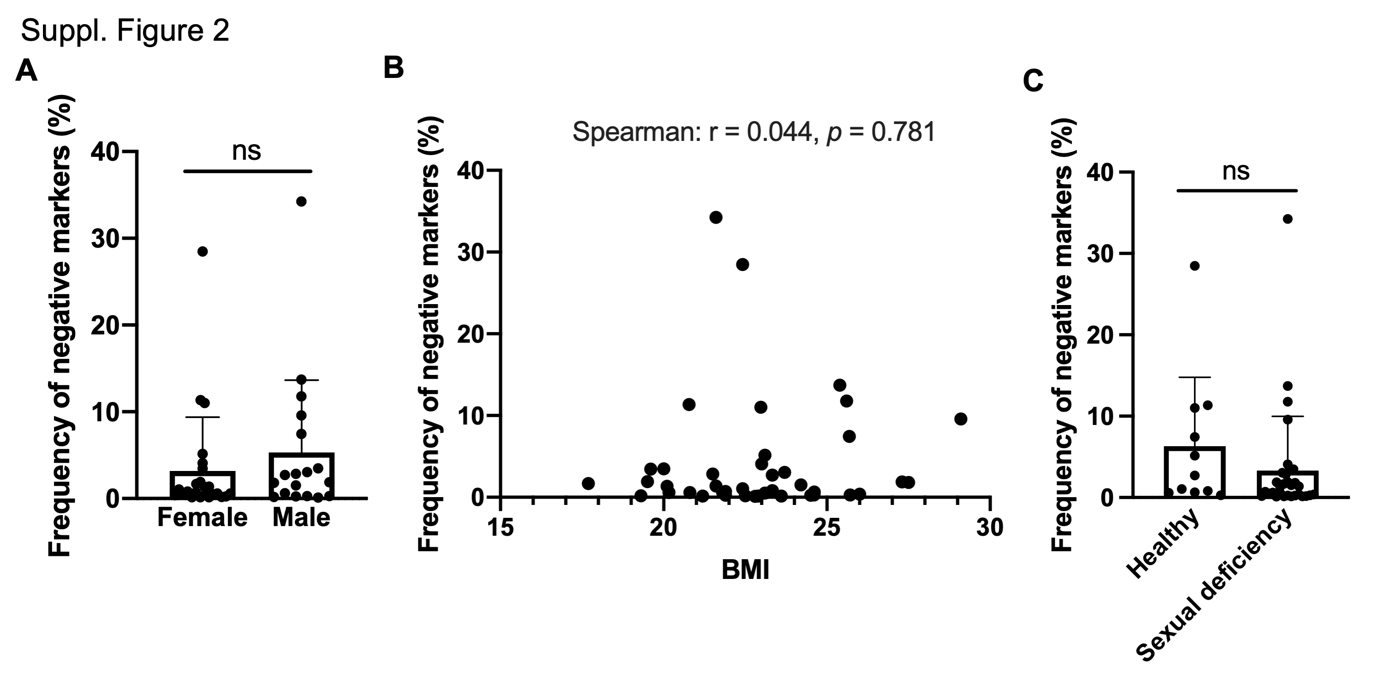


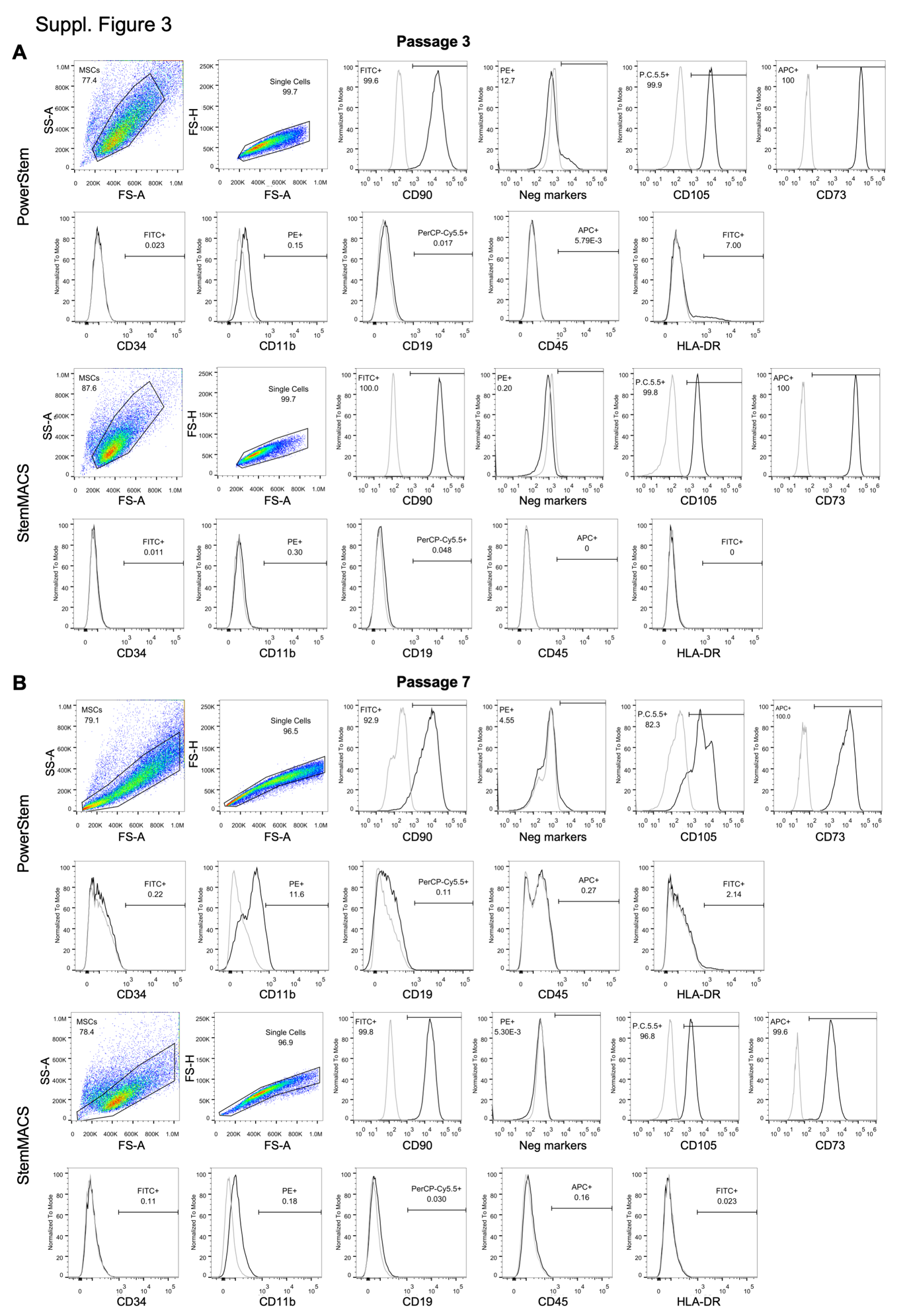


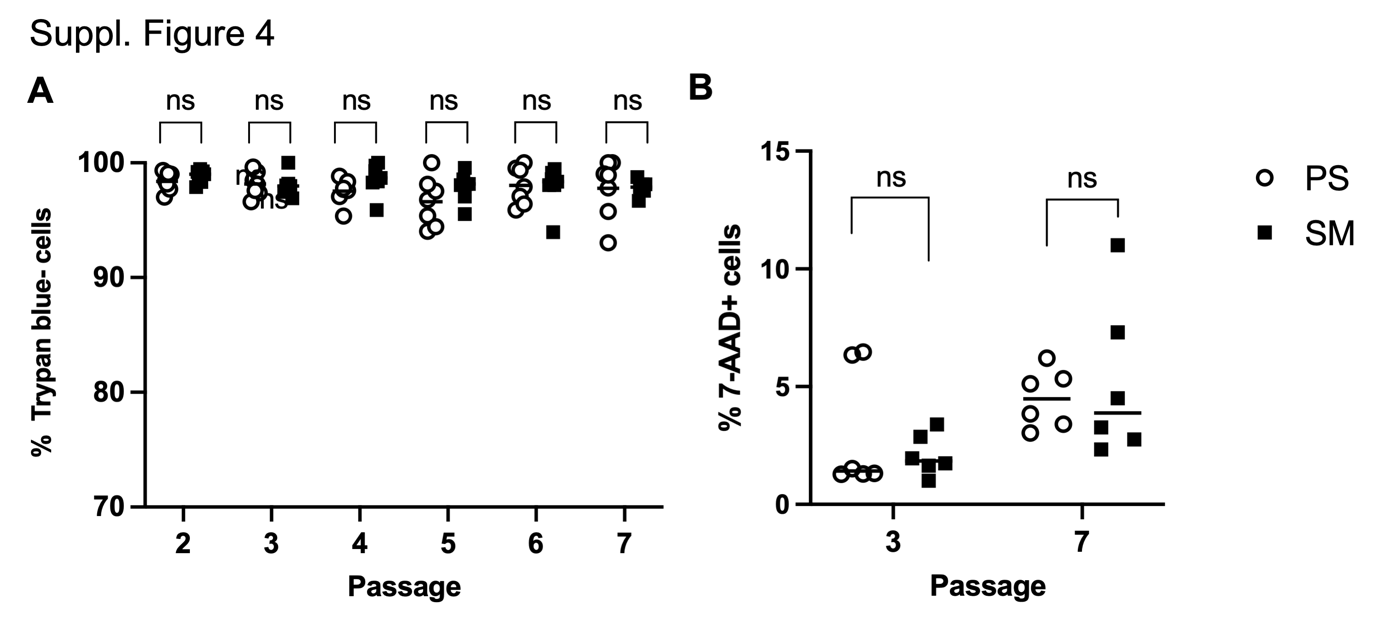

Supplement: Supplementary file 1 — Supplementary file1 (DOCX 15.1 MB) [file 12015_2021_10242_MOESM1_ESM.docx]
